# Temporal modulation of duodenal microbiota in dairy cows: effects of dietary shift from high forage to high concentration

**DOI:** 10.3389/fvets.2025.1551327

**Published:** 2025-04-04

**Authors:** FuWei Wang, Biao Xie, Hongjin Ji, Jianmin Xia, Yangyi Hao, Zhijun Cao, Wei Wang, Min Gao, Shengli Li, Kailun Yang

**Affiliations:** ^1^College of Animal Science, Xinjiang Agricultural University, Ürümqi, China; ^2^State Key Laboratory of Animal Nutrition and Feeding, College of Animal Science and Technology, China Agricultural University, Beijing, China; ^3^State Key Laboratory of Reproductive Regulation and Breeding of Grassland Livestock, Inner Mongolia University, Hohhot, China

**Keywords:** duodenal microbiota, dairy cattle, circadian rhythm, dietary regimes, forage-to-concentration ratio

## Abstract

**Introduction:**

The duodenum and its resident microbiota play crucial roles in the process of nutrient digestion and absorption. However, the temporal dynamics of duodenal microbiota in response to different dietary regimes remain are not yet fully understood. The aim of the present study was to explore the effects of high-forage (HF) and high-concentration (HS) diets on the circadian rhythm variation of duodenal fermentation and microbial communities in dairy cattle.

**Methods:**

Six duodenum-cannulated Holstein dairy cows were assigned to HF and HS diets according to a crossover design with two periods, with each period lasting 21 d (18 d for adaptation, 3 d for sampling). Duodenal content samples were collected at six time points (07:00, 11:00, 15:00, 19:00, 23:00, and 03:00) for the analysis of volatile fatty acids (VFA) and characterization of microbial characterization. The times of 11:00, 15:00, and 19:00 were recorded as the light phase and 23:00, 03:00 and 07:00 were recorded as the dark phase.

**Results:**

The results showed that TVFA displayed a significant circadian rhythm following the introduction of the HS diet (*p* < 0.01). The concentration of TVFA (*p* < 0.01) and acetate (*p* < 0.01) were significantly higher during the light than the dark phase, regardless of diet type. PERMANOVA analysis revealed that diet and diet × time interaction strongly influenced the composition of duodenal microbiota. The relative abundance of Lachnospiraceae_ND3007_group showed a positive correlation with the propionate proportions under the HS diet during the light phase, while the HS diet significantly increased the abundance of *Bifidobacterium* and *norank_f__Lachnospiraceae*.

**Conclusion:**

These findings provide novel insights into diet-dependent circadian regulation of duodenal fermentation in dairy cattle.

## 1 Introduction

Dairy cattle’s relationship with humans arises from their ability to convert human-indigestible carbohydrates and nitrogen into high quality protein in the form of meat and milk (1). The ability of dairy cattle to transform vegetation into humans metabolizable protein and energy is primarily attributed to the microorganisms inhabiting their intestinal tract through alimentary and endogenous trophic systems (2). In addition, the interaction between intestinal microorganisms and the host plays a pivotal role in regulating physiological processes, including feed conversion efficiency, milk production, health, immunity, and nutrition metabolism (3–5). Therefore, elucidating the dynamics of intestinal microbiota and their regulatory mechanisms is crucial to improving feed efficiency and dairy cattle health. For decades, most research has focused on the composition and function of the ruminal microbiome, which is a primary organ in dairy cattle responsible for the nutrient degradation and digestion in dairy cattle (2). However, the current understanding of the microbial community in the small intestine of dairy cows and its impact on host health and productivity is still limited. Importantly, the small intestine and its microbiota play a pivotal role in shaping the health and productivity of dairy cattle (6). Thus, comprehensive analysis of the microbial composition and functional in the small intestine is essential for advancing dairy cattle production efficiency.

The duodenum, a critical segment of the small intestine, is primary for nutrient digestion and absorption (7). In dairy cattle, the pH of the digesta increases rapidly as it passes from the rumen to the duodenum, but the microbial diversity gradually decreases, with a count of around 10^9^ copy/g (8), mainly of the phylum *Firmicutes* (9). The microbiota in the duodenum serves an essential role in the metabolism of macronutrients and vitamins (10). However, our knowledge about microbial function and communities in the duodenum remains limited.

Previous study indicated that diet structure is an essential factor affecting the composition and function of the gastrointestinal microbial community in dairy cattle (11). In dairy cattle production, high-concentration diet was typically introduced to enhance milk production. Nevertheless, high-concentration diet in dairy cattle often result in the accumulation of undigested starch and volatile fatty acids (VFAs) in the duodenum (12), leading to excessive fermentation and disruptions in the intestinal bacterial community. Moreover, studies have demonstrated that lower concentrations of dietary forage can increase the abundance of opportunistic pathogens, such as *Clostridium perfringens* type A (13), and significantly reduce the enzymatic activity associated with hemicellulases (GH10, GH11 and GH54) and cellulases (GH1, GH44 and GH45) (14). Additionally, rhythmic feeding induces periodic changes in environmental conditions within the intestine, including temperature, pH, nutrient availability, and peristalsis. These fluctuations promote the proliferation of microorganisms adapted to specific conditions at different times, driving rhythmic shifts in the composition of the intestinal microbiota. Evidence indicates that the biogeography, composition, and functions of the intestinal microbiota, along with its metabolites, exhibit pronounced diurnal variations (15, 16). However, to date, no data have been reported that the forage concentration induced rhythmic variation of the duodenum microbiota in dairy cattle. In this aspect, efforts to elucidate whether forage concentration can reshape the duodenal microbiota of dairy cows and how the duodenal microbiota rhythmically responds to meet dietary changes are important. Therefore, the experiment aimed to study the influence of high-forage and low-forage diets on the circadian variation of duodenal bacteria in dairy cattle.

## 2 Materials and methods

The procedures and the management of animals were approved by the China Agricultural University Laboratory Animal Welfare and Animal Experimental Ethical Inspection (AW42504202-1-2).

### 2.1 Animals, diets, and experimental design

Six healthy duodenum cannulated-Holstein dairy cattle with similar days in milking (25.7 ± 7 kg), parity (2.83 ± 0.41), and body weight (751.75 ± 71.10 kg) were selected as experimental animals. The experiment was conducted with two periods, with each experimental period lasting 21 d, of which 18 d were for adaptation and 3 days for sampling. The experimental treatments included two groups: high-forage diet (HF) and high-concentration diet (HS). In period 1, six dairy cattle were fed an HF diet, while in period 2, all dairy cattle were switched to the HS diet. The formulation and nutritional components of the experimental diet are shown in Table 1.

**Table 1:** Ingredients and nutritional composition of the experimental diet.

Items	Groups	
	HF	HS
Ingredients, %DM		
Corn silage	35.87	31.50
Alfalfa hay	16.59	5.70
Oat hay	7.62	2.43
Optigen	0.49	1.01
Rapeseed meal	1.91	3.93
Soybean meal	14.94	14.57
Glucose	1.10	2.25
Corn	14.60	29.98
Calcium fatty acid	1.59	3.26
Limestone	0.74	0.77
NaHCO_3_	0.63	0.63
Salt	0.61	0.61
Premix	3.30	3.36
Chemical composition, %DM		
DM	52.34	53.47
CP	15.54	15.30
NE_L_, Mcal/kg	1.57	1.65
Starch	21.01	28.79
NDF	38.21	29.39
ADF	20.51	14.38

NE_L_, net energy for lactation; calculated value using the equation from NASEM (2021).

HF, High forage diet; HS, High concentration diet.

Dairy cattle were housed in individual pens equipped with feed troughs and had ad libitum access to fresh drinking water. The animals were fed total mixed ration (TMR) three times daily at 07:00, 15:00, and 19:00 with a daily feed residue of 2 to 5%. The milk of dairy cattle was collected twice daily at 06:30 and 18:30. The feed samples were collected weekly during each sampling period and stored at −20°C for moisture and chemical composition analysis.

### 2.2 Sampling and measurement

#### 2.2.1 Experimental diet nutrient

The experimental feed samples were freeze-dried for 96 h using a freeze dryer (LGJ-12; Beijing Songyuan Huaxing Technology Development Co. Ltd., Beijing, China). The feed samples were milled using a grinder (FW177, Tianjin Taisite Instrument Co., Ltd., Tianjin, China), and then all samples of feed were passed through a 40-mesh sieve. The dry matter (DM) and crude ash of feed samples were determined according to the methods 930.15, 942.05 and 981.10 described in AOAC (17) respectively. The content of N in all feed samples was analyzed according to AOAC (17) using the method No. 984.13, and crude protein (CP) was calculated as N × 6.25. The organic matter (OM) of feeds and feces was calculated by DM minus crude ash. The neutral detergent fiber (aNDF) and acid detergent fiber (ADF) contents in feeds were determined following the method of Van Soest et al. (18) in an Ankom A200i Fiber Analyzer (Ankom Technology Co., NY, USA). Heat-stable α-amylase and sodium sulfite were used in the NDF analyzed. Total starch in feed sample was measured according to the procedures of Hall et al. (19).

#### 2.2.2 Duodenum fermentation parameters

During each sampling period, before morning feeding, two parts of the duodenum fluid were collected from each dairy cow at 07:00, 11:00, 15:00, 19:00, 23:00 and 03:00. The pH of the duodenum fluid sample was immediately measured using a portable pH meter (model 8601, AZ Instruments Co. Ltd., China). One part of the sample was centrifuged at 12,000 rpm for 10 min, after which the supernatant was collected and preserved at −20°C for VFA concentration analysis via gas chromatography (GC-8600; Beijing Beifen Tianpu Instrument Technology Co., Ltd., Beijing, China). Next, another part of homogenized duodenum fluid was frozen in liquid nitrogen for further 16S rRNA sequencing according to the method described by Qin (20).

#### 2.2.3 Duodenum microbiota 16S rRNA gene sequencing

Bacterial DNA of all duodenum fluid samples was extracted using the E.Z.N.A.^®^ soil DNA Kit (Omega Bio-tek, Norcross, GA, USA) according to the manufacturer’s instructions. The quality of library was determined in NDrop2000 (Thermo Scientific, USA) using 1% agarose gel electrophoresis. The V3–V4 region of the bacterial 16S rRNA gene was amplified using a PCR instrument (ABI GeneAmp^®^ 9700) according to the 341F (5′- CCTACGGGNBGCASCAG-3′) and 805R (5′- GACTACNVGGGTATCTAATCC-3′) primer pair (21). The paired-end sequencing was performed on the Illumina MiSeq PE300 platform (Illumina, San Diego, USA) following the standard protocols of Majorbio Bio-Pharm Technology Co., Ltd. (Shanghai, China). The FASTQ files were processed and merged, and the raw data was subjected to quality filtering, denoising, merging, and amplicon sequence variants (ASVs) calling using the DADA2 plugin in QIIME2 (v2019.7) (22, 23). The ASVs were generated by clustering sequences at 100% similarity, and taxonomic classification for each ASV was performed using a Naive Bayes classifier trained on the SILVA database (24). The bacterial abundance and homogeneity were assessed using the alpha diversity metrics in the R vegan package. Weighted principal coordinates analysis (PCoA) based on unifrac was utilized to illustrate the differences in gut microbial community structure. Co-occurrence networks were constructed using the Spearman correlation coefficient. Rarefaction curves and alpha diversity indices were calculated using Mothur v1.30.1 (25).

### 2.3 Statistical analysis

The sampling times were grouped into two phases, with 11:00, 15:00, and 19:00 being the light phase and 23:00, 03:00 and 07:00 being the dark phase. Ordination analysis of Bray–Curtis distances between two time or dietary groups was conducted based on taxonomic profiles at the ASV levels. Differences of ASV data between groups were evaluated using the PERMANOVA and ANOSIM tests implemented in the R vegan package (v.2.5–6) with 9,999 permutations. The VFA data were analyzed using the two-tailed t-test of SPSS software (SPSS, IBM, SPSS Statistics, v.25.0) and visualized using the R ggplot2 package. The level of *p* < 0.05 indicates significant differences, while 0.05 ≤ *p* ≤ 0.10 is indicated as a tendency.

## 3 Results

### 3.1 Duodenum fermentation parameters

**Figure 1:**
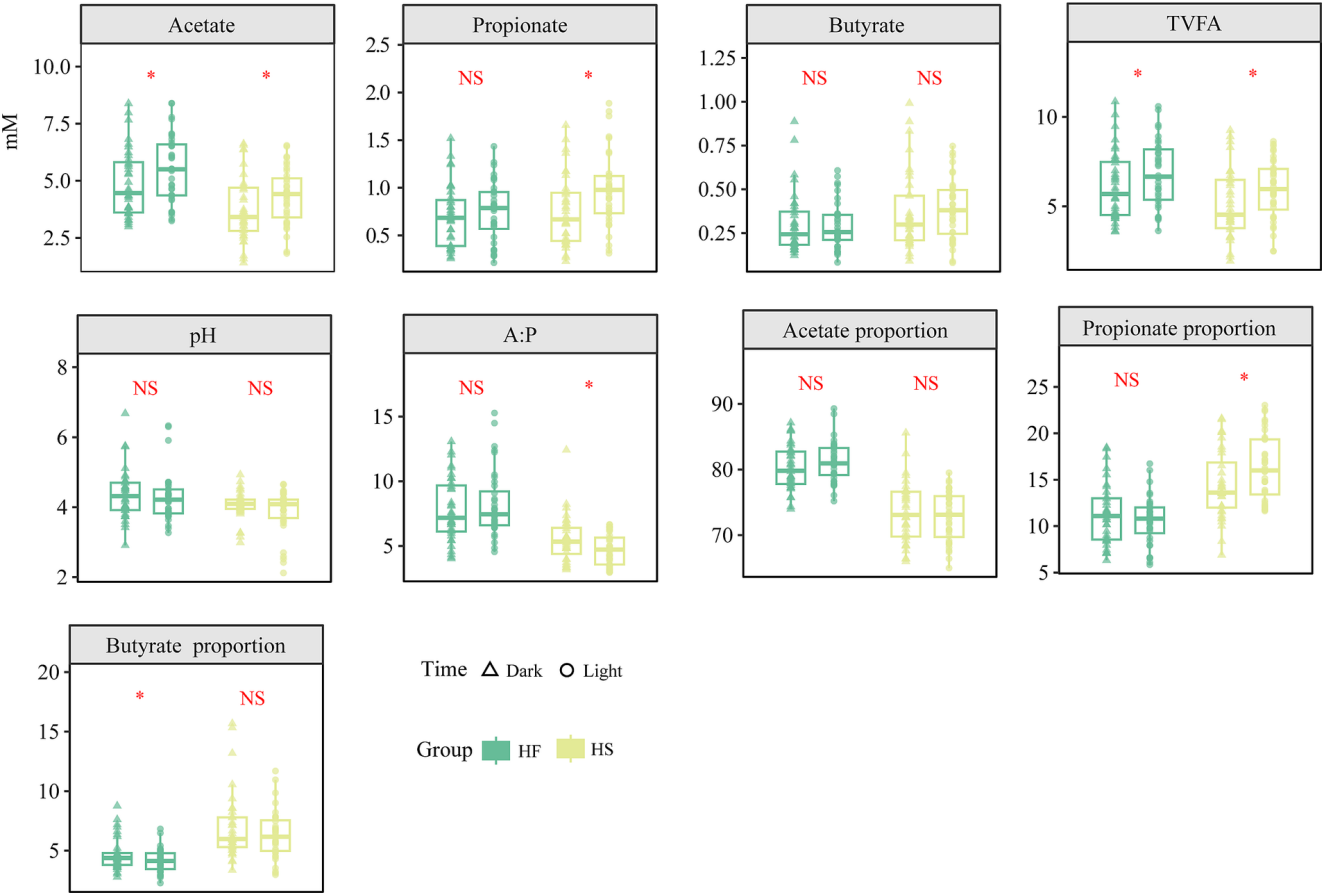
Diurnal dynamic changes in the concentration and ratio of duodenal fermentation parameters in dairy cows fed high-fiber and high-starch feeds. TVFA, Total volatile fatty acids; HF, high-fiber group; HS, high-starch group. The asterisk (*) indicates a statistically significant difference (*p* < 0.05).

To explore the different fermentation indicators in the duodenum after the HF and HS introduction during various time points, we compared the VFA differences across diets at 6 time points. The results indicated that acetate constituted approximately 70–90% of the total VFA in the duodenum, while the concentration of TVFA and acetate was significantly increased in light compared to dark regardless of whether the HF or HS diet was provided (*p* < 0.05). Similarly, the concentration and proportion of propionate was not significantly changed after the HF diet provided, significantly elevated during the light phase (*p* < 0.05) after HS diet was introduced (Figure 1).

### 3.2 Duodenum microbiota profiles

**Figure 2:**
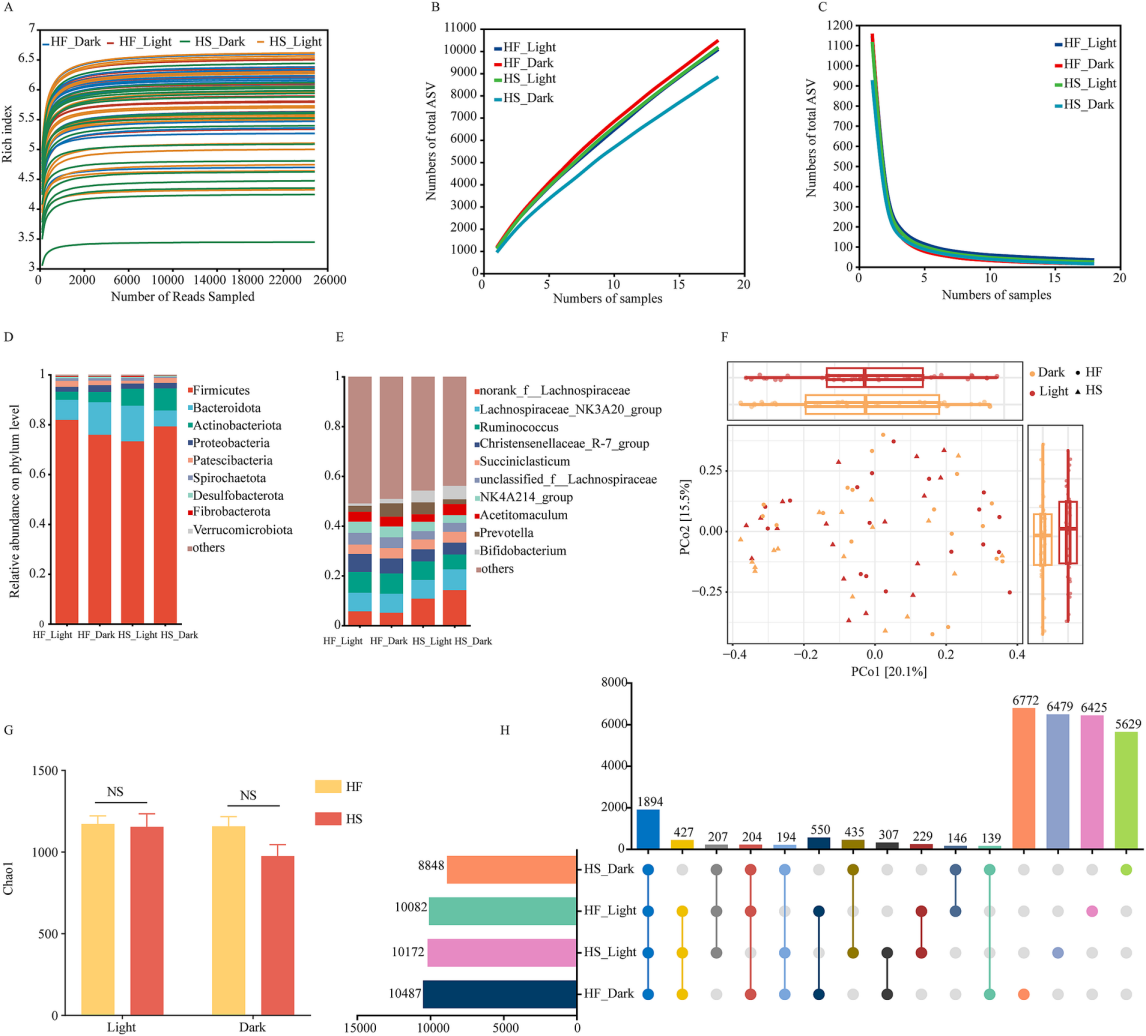
Diurnal dynamic changes of duodenal microbiota in dairy cows fed high-fiber and high-starch feeds. **(A)** Rarefaction analysis of bacteria at the ASV level. **(B,C)** Pan/core analysis at ASV level, *X* and *Y*-axis, respectively, represent the number of observed samples and the number of ASV shared by all samples in a grouping category; pan ASV and core ASV, respectively, represent the union number of common ASV and the intersection number of common ASV. **(D)** The relative abundances of duodenal bacteria at the phylum level. **(E)** The relative abundances of duodenal bacteria at the genus level. **(F)** Principal component analysis (PCA) based on ASVs among different groups. **(G)** The dynamic change of Chao1 indices of bacterial communities in dairy cows fed high-fiber and high-starch feeds. **(H)** Comparison of the duodenal microflora of cows fed a high-starch and high-fiber diet during the light and dark phase. HF, high fiber group; HS, high-starch group. Light phase: 11:00, 15:00 and 19:00; Dark phase: 23:00. 3:00, 7:00.

In total, 349,0036 high-quality sequences were obtained, with over 48,472 for each sample and an average length of 411 bp for each sequence. The high-quality reads were clustered into 3,0037 ASVs at 100% similarity. All rarefaction curves of the richness index on ASV level tend to reach a plateau at 10,000 reads, indicating that the depth was saturated (Figure 2A). In Pan/core analysis, with an increasing number of samples, the total microbiota species richness increased, while the core gut microbiota species appeared to reach a plateau (Figures 2B,C). At the phylum level, the taxonomic analysis of the reads identified more than 10 different fecal bacteria, of which *Firmicutes* and *Bacteroidota* accounting for 77.58 and 10.43% of the total reads, respectively (Figure 2C), and at the genus level, more than 28 different fecal bacteria were identified, of which *norank_f_Lachnospiraceae*, *Lachnospiraceae_NK3A20_group*, *Ruminococcus* and *Acetitomaculum* were the four prevalent bacteria and accounted for 9.00, 7.74, 7.48 and 3.89% of the total reads, respectively (Figures 2D,E). The Bray-Curtis distance-based β-diversity analysis of duodenal microbiota did not show clear separations at light and night regardless of whether the HF or HS diet was provided, while HF or HS diet did not significantly alter the α-diversity (Chao1) during light or night (Figures 2F,G). Among all the detected ASV, the abundance of duodenal microorganisms was higher at night following the HF diet, whereas it was higher during the light after the HS diet introduction (Figure 2H).

### 3.3 Duodenum microbiota co-occurrence patterns

**Figure 3:**
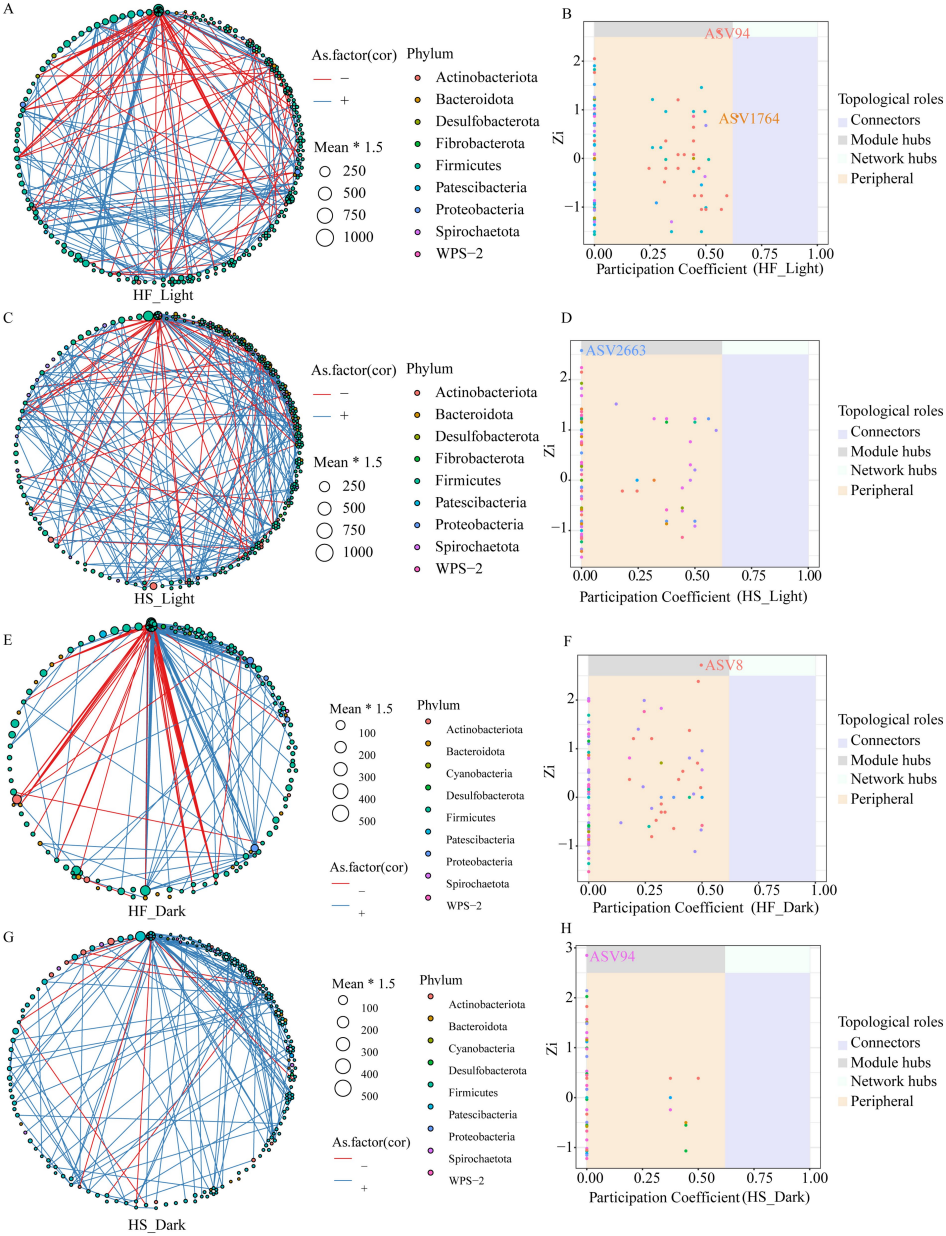
Co-occurrence network of ASVs, and distribution of ASVs based on their network roles among different groups. Nodes represent ASVs, and the color of connection lines between two nodes represents a positive (red) or a negative (blue) correlation (Spearman’s correlation, *p* <  0.05). Distributions of keystone taxa corresponding to their within-module connectivity (Zi) and among-module connectivity (Pi). HF, high-fiber group; HS, high-starch group. Light phase: 11:00, 15:00 and 19:00; Dark phase: 23:00. 3:00, 7:00.

We performed the time-based network analysis to further evaluate the potential microbial modules and identify the keystone bacteria following the diet and time treatment (Figures 3A–H). The microbial co-occurrence networks revealed distinct patterns under different dietary treatments and sampling times. The results indicated that negative correlations dominated the networks, with stronger connectivity during the light phase following the HS diet compared to the HF diet. Network complexity was generally reduced during the dark regardless of whether the animal was fed HF or HS diet. Notably, two network hub ASV including ASV94 (*g__Family_XIII_UCG-001*) and ASV1764 (*f__Lachnospiracea*e) were detected during the light phase after the animals were fed the HF diet, while one hub ASV, ASV8 (*g__Lachnospiraceae_NK3A20_group*) was detected during the dark phase. ASV2663 (*g__Eubacterium_ruminantium_group*) and ASV94 (*g__Family_XIII_UCG-001*) were identified as hub microbiota during the light and dark, respectively, after the animals were fed HS diets.

### 3.4 Diurnal response of duodenal microorganisms to HF and HS diets

**Figure 4:**
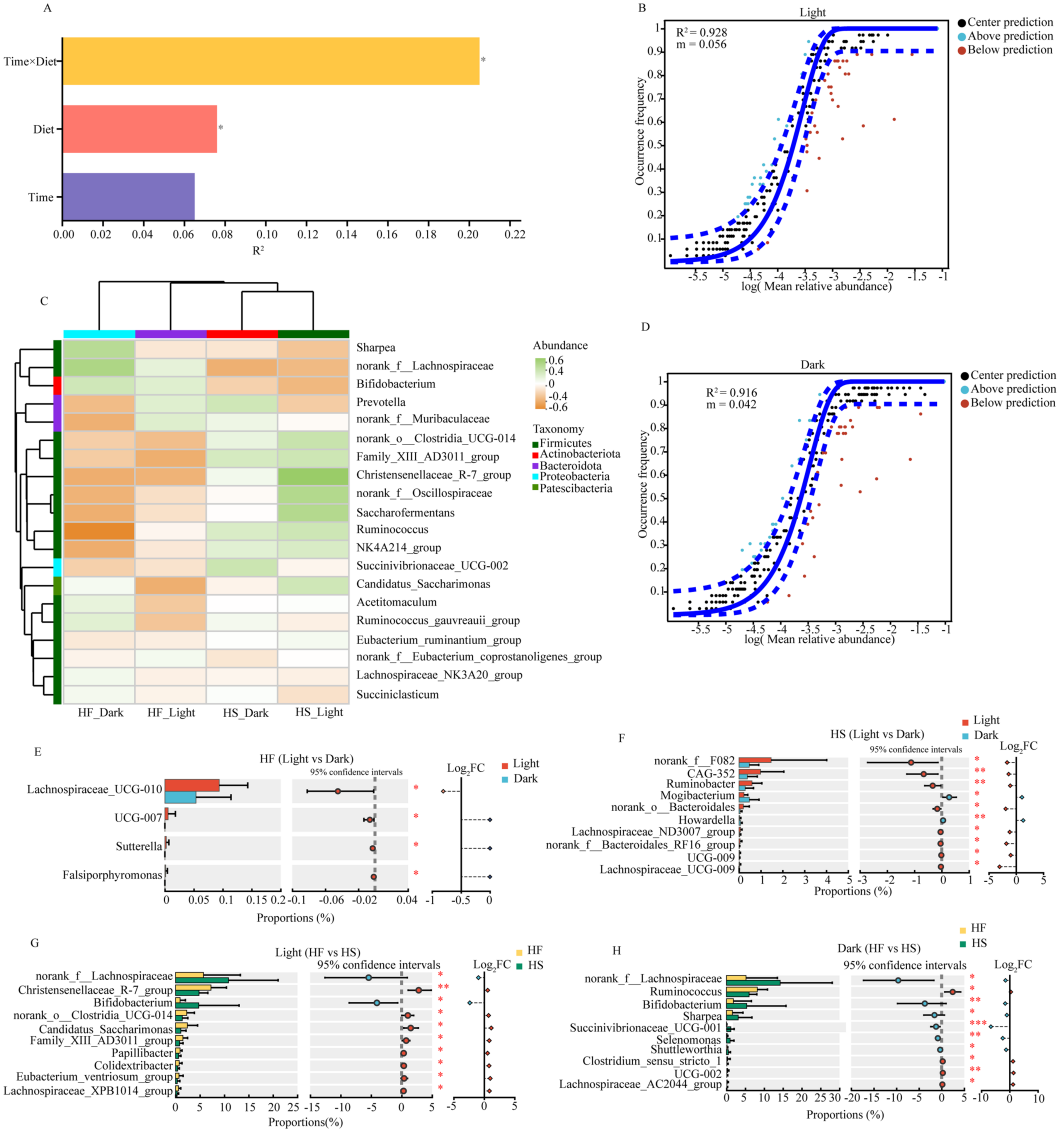
Impact of diet and temporal factors on duodenal microbial fluctuations. **(A)** PERMANOVA analysis of the diet and time influencing the gut microbiota. Only the significant differences were marked (*P < 0.05, **0.01< *P* < 0.05, ***P < 0.001). **(B)** near-neutral model of duodenal microbiota at light phase. **(C)** The heat map shows the correlation between different groups and the top 20 abundant microorganisms. **(D)** near-neutral model of duodenal microbiota at dark phase. **(E)** Significantly different microbiota after the dairy cattle fed HF diet during the light and dark phase. **(F)** Significantly different microbiota after the dairy cattle fed HS diet during the light and dark phase. **(G)** Significantly different microbiota during the Light phase after the dairy cattle fed HF or HS die. **(H)** Significantly different microbiota during the Dark phase after the dairy cattle fed HF or HS die. HF: high-fiber group; HS: high-starch group. Light phase: 11:00, 15:00 and 19:00; Dark phase: 23:00. 3:00, 7:00.

PERMANOVA analysis revealed that the interaction between time and diet (*R*^2^ = 0.21, *p* = 0.37) exhibited the strongest influence on duodenal microbiota, followed by independent effects of diet (*R*^2^ = 0.08, *p* = 0.43) (Figure 4A). The neutral community model displayed high goodness of fit for both light (*R*^2^ = 0.928, *m* = 0.056) and dark (*R*^2^ = 0.916, *m* = 0.042) periods (Figures 4B,D). In addition, distinct microbial patterns across different treatments were observed, with notable variations in *Ruminococuss*, *Candidatus_Saccharimonas*, and *Family_XIll_AD3011_group* (Figure 4C). The abundance of microbiota was higher during the light than during the dark under both HF and HS diets, with more differentially abundant microbiota observed in the HS diet group. After the introduction of the HF diet, the relative abundance of *Lachnospiraceae_UCG-010, UCG-007*, and *Sutterella* was significantly increased during the light compared to dark (Figure 4E). Meanwhile, under the HS diet, the relative abundance of *norank_f_F082*, *CAG-352*, *Ruminobacter*, *Mogibacterium*, *norank_o_Bacteroidales*, *Howardella*, and *Lachnospiraceae_ND3007_group* was significantly increased during the light phase (Figure 4F). Feeding the HF diet significantly increased the abundance of duodenal microbiota, including *norank__f__Lachnospiraceae*, *Christensenellaceae_R-7_group*, *Bifidobacterium*, *norank__o___Clostridia_UCG-014*, *Candidatus_Saccharimonas*, *Family_XIII_AD3011_group* and *Papillibacter* during the light phase (Figure 4G), but significantly decreased the relative abundance of *norank__f__Lachnospiraceae*, *Ruminococcus*, *Bifidobacterium*, *Sharpea*, *Succinivibrionaceae_UCG-001*, *Selenomonas*, *Shuttleworthia* and *Clostridium_sensu_stricto_1* during the dark phase (Figure 4H).

### 3.5 Rhythmic alterations in the metabolic cascade of the duodenal microbiome by high of low forage diet

**Figure 5:**
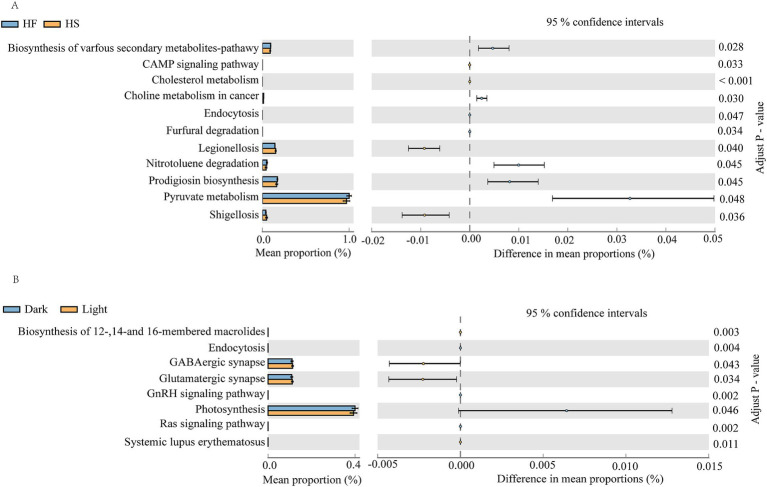
PICRUSt2 functional prediction analysis of duodenal microbes. **(A)** Comparison of duodenal microbial functions in cows fed HF and HS diets. **(B)** Diurnal variation of gastrointestinal microbial function in dairy cattle. F, high-fiber group; S, high-starch group. Light phase: 11:00, 15:00 and 19:00; Dark phase: 23:00. 3:00, 7:00.

The KEGG pathway analysis using PICRUSt 2.0 showed that after HF diet introduction, the pathways involved in the biosynthesis of various secondary metabolites-pathway, choline metabolism in cancer, nitrotoluene degradation, endocytosis, furfural degradation, prodigiosin biosynthesis and pyruvate metabolism were significantly increased, but the CAMP signaling pathway, cholesterol metabolism, legionellosis and shigellosis were significantly decreased relative to the HS group (Figure 5A). Additionally, the function of duodenal microorganisms also changes over time. During the light phase, the function of biosynthesis of 12-,14-and 16-membered macrolides, GABAergic synapse, glutamatergic synapse and systemic lupus erythematosus was significantly upregulated, while the endocytosis, GnRH signaling pathway, photosynthesis and Ras signaling pathway was significantly downregulated compared to the dark phase (Figure 5B).

### 3.6 Correlations between key bacteria fermentation parameters

**Figure 6:**
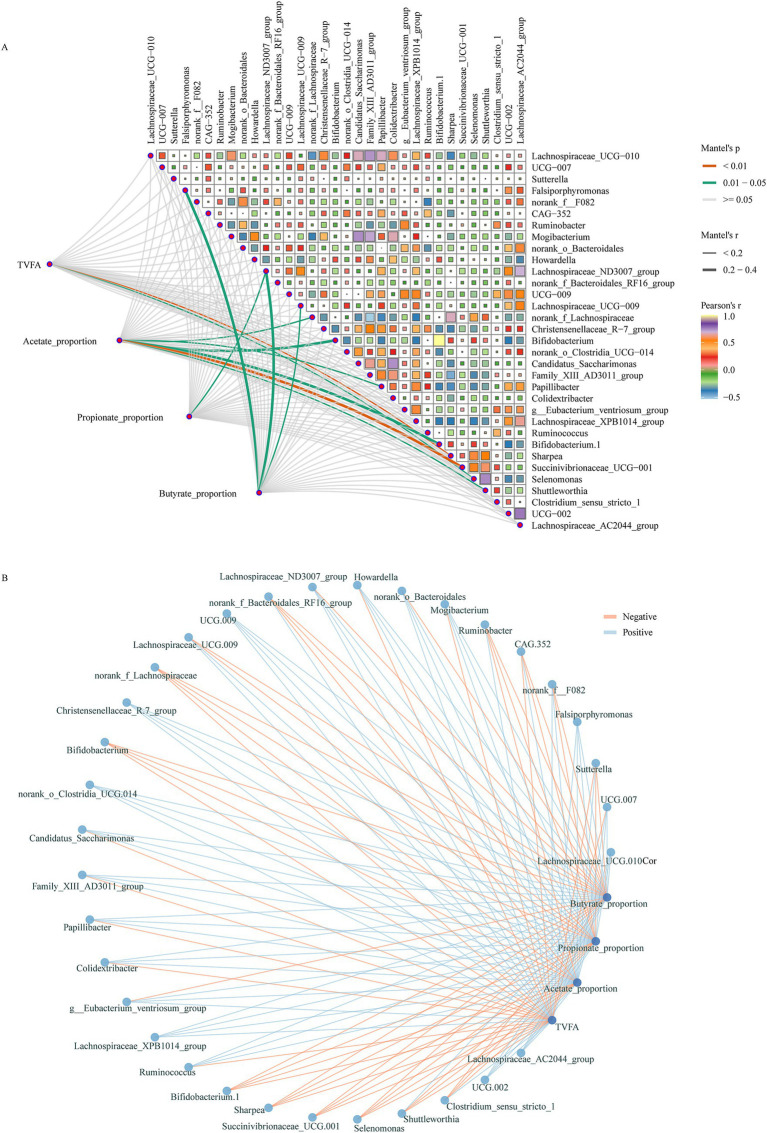
Correlations between key bacteria and fermentation parameters. **(A)** Relationships between different groups enriched bacteria and fermentation parameters. The correlation was determined by the Mantel test. The edge color and width denote the statistical significance and the correlation coefficient (Mantel r). **(B)** Network plots for the different groups enriched bacteria and fermentation parameters, the lines represent the relationships (red for positive and green for negative).

The correlations between key bacteria and fermentation parameters are demonstrated in Figure 6. The results indicated that the TVFA was significantly negatively correlated with the *sharpea* and *Selenomonas*, which were significantly increased after HS diet introduction during the dark phase. In addition, bacteria enriched in animals fed HS diet such as *Bifidobacterium*, *norank_f__Lachnospiraceae* and *Succinivibrionaceae_UCG-001* were significantly negatively correlation with the molar proportions of acetate. The bacteria enriched during the light phase after HS diet introduction including *Lachnospiraceae_ND3007_group* were significantly positively correlated with the molar proportions of propionate and negatively correlated with the molar proportions of butyrate. Moreover, the bacteria of *Lachnospiraceae_UCG−009,* which increased in the light phased after the animal was fed HS diet were significantly negatively correlated with the molar proportions of butyrate (Figures 6A,B).

## 4 Discussion

The duodenum of dairy cows is the primary for nutrient digestion and absorption, where its fermentation pattern and microbiota play crucial roles in digestion, metabolism, and immune function (26). Herein, we demonstrate that the duodenal microbiota of dairy cows exhibits a diurnal rhythm and is primarily influenced by the ratio of concentrate to forage. Interestingly, the TVFA concentrations in the duodenum were higher during the light than during the dark whether the animals were fed high-forage or low-forage diets, but the duodenal microbial α- diversity and β-diversity did not alter with diet or time of day. Nevertheless, the duodenal microbial abundance was higher during the light and lower during the dark after dairy cattle fed low-forage diets compared to high-forage diets.

Fluctuations in the intestinal short-chain fatty acids are closely related to intestinal homeostasis and energy metabolism (27). The distinct patterns of VFA production during light and dark phases under different dietary conditions reveal the complex interactions between circadian rhythms and dietary composition in ruminant gastrointestinal fermentation. In the present experiment, the concentration of acetate was significantly increased during the light phase, regardless of whether HF or HS diets were provided. The VFAs in the duodenum originate mainly from feed fermentation in the rumen (28). Thus, the increase of acetate related to the rumen fermentation and may be attributed to several factors, including the enhanced feeding behavior and ruminal microbial enzyme activity during the light period (29), and the synchronization of ruminal bacterial metabolic activities with the host’s circadian clock (30). Consequently, the results in the present experiment showed that the concentration of TVFA was also significantly increased during the light phase, which can be primarily explained by the increased acetate levels, as acetate typically accounts for the majority of TVFA. Moreover, the concentration and proportion of propionate was significantly increased during the light phases under the HS diet, which can be attributed to the high-starch diets promote the growth of propionate-producing bacteria activity (31). This result is consistent with the findings of Santra et al., who found that feeding high concentrate diets could increase the concentration and proportion of propionate in the rumen (32). These temporal variations in VFA profiles influenced by diet not only reflect the dynamic nature of ruminal and duodenal fermentation but also suggest potential opportunities for optimizing feeding strategies to enhance ruminant feed utilization efficiency and ultimately improve the animal growth performance.

We next observed that the duodenal microbiota was less affected by time, which was further verified by the fact that difference in duodenal microbiota was better explained by dietary regime than circadian rhythm. Of the taxonomic populations, the predominant phyla were *Firmicutes* and *Bacteroidota* in the duodenal microbiota, which accounted for approximately 88% of the total bacterial population. This finding aligns with previous studies on the intestinal microbiome (33). At the genus level, the dominant microbiota was *norank_f_Lachnospiraceae*, *Lachnospiraceae_NK3A20_group*, *Ruminococcus*, and *Acetitomaculum*, suggesting their crucial roles in duodenal fermentation and nutrient metabolism. Notably, it was reported that members of the *Lachnospiraceae* family are particularly known for degrading complex carbohydrates and producing short-chain fatty acids, which serves as the primary energy source for colonocytes and maintains intestinal barrier function (34). Furthermore, the results in the present experiment indicated that the duodenal microbiota exhibits temporal variations that are significantly influenced by dietary composition. In detail, the abundance of duodenal microbiota exhibited a consistent diurnal rhythm with higher levels during the light phase irrespective of dietary forage ratio, indicating that microbial activity was stronger during the day, which coincided with elevated concentrations of acetate and TVFA in the duodenal. Additionally, we found that the relative abundance of *norank_f__Lachnospiraceae* and *Bifidobacterium* was significantly increased after HS diet introduction whether during the light or dark phase. Similarly, the relative abundance of *Lachnospiraceae_ND3007_group* and *Lachnospiraceae_UCG-009* was significantly increased during the light than dark phase after HS diet introduction. This shift in microbial composition enhances the metabolic capacity for starch degradation, as *Lachnospiraceae* possess diverse glycoside hydrolases that efficiently break down dietary polysaccharides into readily absorbable nutrients (35). Meanwhile, members of the *Lachnospiraceae* family can generate propionate via the succinate and propanediol pathways, which may be a crucial reason for the increased proportion of propionate during the light phase after the HS diet was introduced. Research has also shown that *Bifidobacterium* enhances nutrient absorption through vitamin synthesis, particularly B vitamins that serve as essential cofactors in various metabolic pathways, while also strengthening the intestinal barrier through the production of acetate and lactate (36). These results suggest that feeding a low-forage diet may have a positive effect on the hindgut health and immunity of dairy cows. Notably, our results demonstrated that under the HF diet, there was no consistent pattern in microbial changes between light and dark phases. This inconsistent diurnal rhythmicity might be attributed to the complex nature of fiber degradation that requires diverse microbial communities with different metabolic capabilities, leading to asynchronous growth patterns (37). Another reason for this result may be related to the sequential degradation of dietary fiber components, which may lead to temporal ecological niche partitioning between different bacterial groups, where early colonizers and secondary degraders show different temporal dynamics (38).

## 5 Conclusion

Different forage-to-concentrate ratios significantly affected the diurnal patterns of duodenal fermentation and microbiota in dairy cattle. The HS diet induces greater temporal variations in VFA profiles and microbial composition compared to the HF diet. The abundance of duodenal microbiota and VFA concentrations exhibit consistent diurnal rhythms, with higher levels during the light phase, particularly under the HS diet. This work provides insights into diet-dependent circadian regulation of duodenal fermentation, which has important implications for optimizing feeding strategies to enhance nutrient utilization in dairy cows. Moreover, these findings have direct applications for dairy farm management, such as that concentrate feeds could be strategically delivered during the light phase (11:00–19:00) to align with peak microbial activity, and dietary starch content could be distributed throughout the day to optimize nutrient utilization as well as feeding frequency could be adjusted to synchronize with natural peaks in microbial metabolism. Future research should focus on exploring the molecular mechanisms underlying the diet-microbiota-host interactions and investigating the long-term effects of these temporal variations on dairy cattle health and productivity.

## Data Availability

The raw 16s rRNA sequencing data of the duodenal bacteria were deposited in NCBI (PRJNA1224208).
